# Clinical characteristics of chronic obstructive pulmonary disease patients with superoptimal peak inspiratory flow rate

**DOI:** 10.1038/s41598-024-65085-2

**Published:** 2024-07-03

**Authors:** Taeyun Kim, Ji-Yong Moon, Hye Yun Park, Youlim Kim, Chin Kook Rhee, Chang Youl Lee, Joo Hun Park, Yong Bum Park, Richard Russell, Kwang Ha Yoo, Seung Won Ra

**Affiliations:** 1 Division of Pulmonary Medicine, Department of Medicine, Kosin University Gospel Hospital, Kosin University College of Medicine, Busan, Republic of Korea; 2 Department of Internal Medicine, Hanyang University College of Medicine, Seoul, Republic of Korea; 3 Division of Pulmonary and Critical Care Medicine, Department of Internal Medicine, Samsung Medical Center, Sungkyunkwan University School of Medicine, Seoul, Republic of Korea; 4 Department of Internal Medicine, Konkuk University Medical Center, Konkuk University School of Medicine, Neungdong-ro, Gwangjin-gu, Seoul, Republic of Korea; 5 Division of Pulmonary and Critical Care Medicine, Department of Internal Medicine, College of Medicine, Seoul St. Mary’s Hospital, The Catholic University of Korea, Seoul, Republic of Korea; 6 Division of Pulmonary, Allergy and Critical Care Medicine, Department of Internal Medicine, Hallym University Chuncheon Sacred Heart Hospital, Chuncheon-si, Gangwon-do Republic of Korea; 7 Department of Pulmonary and Critical Care Medicine, Ajou University School of Medicine, Suwon, Republic of Korea; 8 Division of Pulmonary, Allergy and Critical Care Medicine, Department of Internal Medicine, Hallym University Kangdong Sacred Heart Hospital, Seoul, Republic of Korea; 9 King’s Centre for Lung Health, King’s College London, London, UK; 10 Division of Pulmonary and Critical Care Medicine, Department of Internal Medicine, Ulsan University Hospital, University of Ulsan College of Medicine, 25 Daehakbyeongwon-ro, Dong-gu, Ulsan, 44033 Republic of Korea

**Keywords:** Superoptimal, PIFR, COPD, Characteristics, Exacerbation, Chronic obstructive pulmonary disease, Outcomes research

## Abstract

Characteristics of chronic obstructive pulmonary disease (COPD) patients with superoptimal peak inspiratory flow rates (PIFR) has not been thoroughly investigated. This study aimed to compare the characteristics between COPD patients with superoptimal PIFR and those with optimal and sub-optimal PIFR. PIFR was measured using In-Check DIAL G16 and categorized into sub-optimal (PIFR lower than that required by the patient’s device), optimal, and superoptimal (peak PIFR ≥ 90 L/min). Considering COPD patients with sub-optimal PIFR as the reference group, analyses were performed to identify PIFR-related factors. Subgroup analysis was performed according to the forced expiratory volume in 1 s (FEV_1_) % of the predicted value (%pred). Among 444 post-bronchodilator-confirmed COPD patients from seven tertiary hospitals in South Korea, 98, 223, and 123 were classified into the sub-optimal, optimal, and superoptimal PIFR groups, respectively. The superoptimal PIFR group were younger, had an increased proportion of males, a higher body mass index, lowest number of comorbidities and less frequent exacerbation in the previous year, as well as the highest forced vital capacity %pred. The adjusted odds ratio for frequent exacerbation in the previous year was lower in the superoptimal PIFR group than in the sub-optimal PIFR group and was more pronounced in patients with an FEV_1_%pred of < 70%. COPD patients with superoptimal PIFR have clinical characteristics different from those patients with the sub-optimal and optimal PIFR. Having a high inspiratory flow may be a favorable trait in COPD.

## Introduction

Chronic obstructive pulmonary disease (COPD) is a chronic respiratory condition characterized by persistent airflow limitation and a significant symptom burden. Inhalers play a crucial role in COPD treatment by delivering bronchodilators and anti-inflammatory medications directly into the lungs^1^. Currently, dry powder inhalers (DPIs), pressurized metered-dose inhalers, soft mist inhalers, and nebulizers are used to deliver a variety of COPD medications^1^. The choice of an inhaler device depends on various factors, including the patient’s ability to effectively use the device, coordination, and their physical abilities. Among them, the inspiratory flow rate is an essential factor when determining the appropriate inhaler type for an individual^1^.

A sub-optimal peak inspiratory flow rate (PIFR) is a common problem and leads to insufficient drug delivery into the lungs to induce effective bronchodilation or other clinical effect. Studies have largely focused on the difference between optimal and sub-optimal PIFR groups^2–5^ and have shown that patients with sub-optimal PIFR are more likely to have advanced-stage disease, older age, and lower lung function compared to patients with optimal PIFR^3^. Sub-optimal PIFR is also related to a shorter time to exacerbation^5^ and readmission^6^. However, the optimal PIFR group in these studies included patients with a PIFR > 90 L/min, which is considered a superoptimal, excessive, or a fast PIFR^7,8^. A recent study in stable COPD patients investigated excessive PIFR at > 90 L/min and showed that the majority of excessive PIFR was observed against low-resistance DPI devices, regardless of age, sex, body mass index (BMI), symptom score, and degree of airflow limitation^8^. This high PIFR group was considered problematic based on a priori premise from the study by Usmani et al.^9^. In that study, fast PIFR resulted in drug deposition, mainly in the upper respiratory tract^9^. However, these data were derived from an aerosol generator and not real patients and devices. Another study revealed that high PIFR in COPD patients who were using DPIs exhibited a more favorable inhalation profile than that associated with low PIFR^10^. Thus, there exists a knowledge gap regarding the association between the severity and degree of PIFR and the clinical characteristics of patients with COPD, especially those with a superoptimal PIFR.

In this context, this multi-center observational study in South Korea aimed to compare the clinical characteristics between COPD patients with superoptimal PIFR and those with optimal and sub-optimal PIFR in real-world clinical setting.

## Results

**Figure 1:**
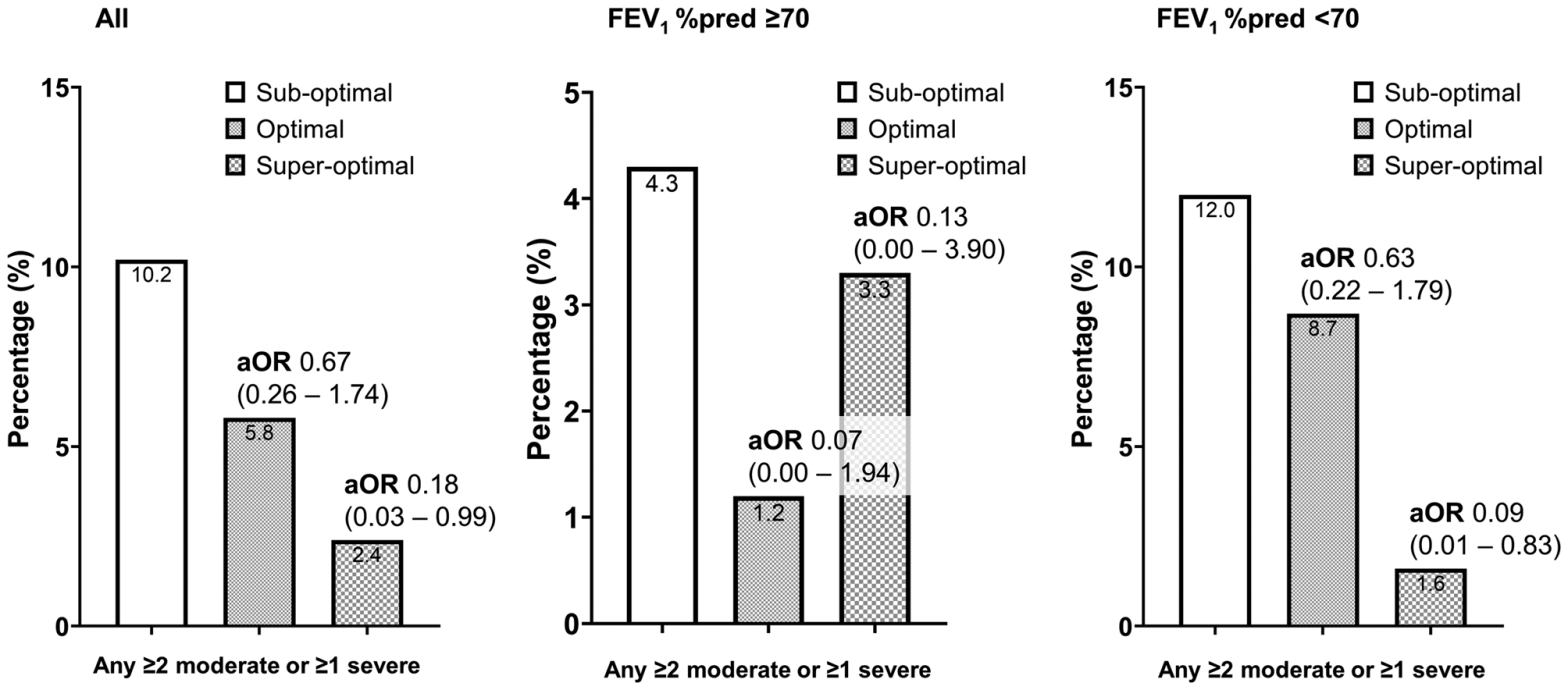
Proportion and adjusted aOR of exacerbations in the previous year (any ≥ 2 moderate of ≥ 1 severe) by PIFR and FEV_1_%pred. *OR was adjusted for age, sex, BMI, smoking status, CAT score, CCI score, and FVC %pred. *aOR* adjusted odds ratio, *BD* bronchodilator, *BMI* body mass index, *CAT* COPD assessment test, *CCI* Charlson comorbidity index, *COPD* chronic obstructive pulmonary disease, *FEV*_*1*_ forced expiratory volume in 1 s, *FVC* forced vital capacity, *%pred* % of the predicted value.

Among the 444 COPD patients using DPI, 98 (22.1%), 223 (50.2%), and 123 (27.7%) were classified into the sub-optimal, optimal, and superoptimal PIFR groups, respectively (Table 1). The superoptimal group consisted of younger patients, higher proportion of males, higher BMI, lower Charlson comorbidity index (CCI) scores, higher forced expiratory volume in one second (FEV_1_) % of the predicted value (%pred), higher forced vital capacity (FVC) %pred, and higher PIFR values compared to the same parameters associated with the sub-optimal group. The proportion of frequent exacerbations in the previous year was significantly lower in the superoptimal PIFR group than in the optimal and sub-optimal groups (Fig. 1, *p* for trend = 0.015).

**Table 1:** Characteristics of COPD patients according to PIFR.

	Sub-optimal (n = 98)	Optimal (n = 223)	Superoptimal (n = 123)	*p*
Age (years)	74.2 (8.1)	71.6 (8.1)*	68.9 (7.3)^†^	< 0.001
Male, n (%)	84 (85.7)	211 (94.6)*	119 (96.9)^†^	0.003
BMI (kg/m^2^)	22.9 (3.2)	23.5 (3.6)	24.4 (2.8)^†^	0.003
Smoking, n (%)				0.024
Never	7 (7.1)	6 (2.7)	3 (2.4)	
Former	77 (78.6)	180 (80.7)	87 (70.1)	
Current	14 (14.3)	37 (16.6)	33 (26.8)	
CAT score	11.8 (7.9)	9.3 (6.9)*	9.9 (7.1)	0.02
CCI score	1.7 (1.4)	1.4 (1.3)*	1.0 (0.9)^†^	< 0.001
FEV_1_%pred^a^	58.2 (16.9)	63.8 (16.3)	69.0 (17.3)^†,‡^	< 0.001
FVC %pred^a^	74.0 (15.7)	80.0 (16.0)*	86.8 (15.8)^†,‡^	< 0.001
Post BD FEV_1_/FVC%	53.5 (12.7)	55.0 (12.5)	55.8 (9.9)	0.36
DLCO %pred (n = 301)	61.0 (22.2)	67.5 (21.5)	70.4 (19.2)^†^	0.019
RV/TLC% (n = 172)	46.0 (11.7)	43.4 (10.5)*	37.6 (10.6)^†^	< 0.001
Highest PIFR (L/min)	41.5 (12.7)	68.6 (10.4)*	105.1 (12.3)^†,‡^	< 0.001
Frequent exacerbation^b^, n (%)	10 (10.2)	13 (5.8)	3 (2.4)	0.051

*COPD* chronic obstructive pulmonary disease, *PIFR* peak inspiratory flow rate, *BMI* body mass index, *CAT* COPD assessment test, *mMRC* modified medical research council, *CCI* Charlson comorbidity index, *FEV*_*1*_ forced expiratory volume in 1 s, *FVC* forced vital capacity, *BD* bronchodilator, *DLCO* diffusion capacity, *RV* residual volume, *TLC* total lung capacity, *% pred* % of the predicted value.

Continuous and categorical variables are presented as means with standard deviations and numbers with percentages, respectively.

^a^The value was obtained in post-bronchodilator spirometry.

^b^Frequent exacerbation was defined as ≥ 2 moderate or ≥ 1 severe exacerbation in the previous year.

*p < 0.017 versus sub-optimal in post-hoc analysis with Bonferroni correction.

^†^*p* < 0.017 versus sub-optimal in post-hoc analysis with Bonferroni correction.

^‡^*p* < 0.017 versus optimal in post-hoc analysis with Bonferroni correction.

**Figure 2:**
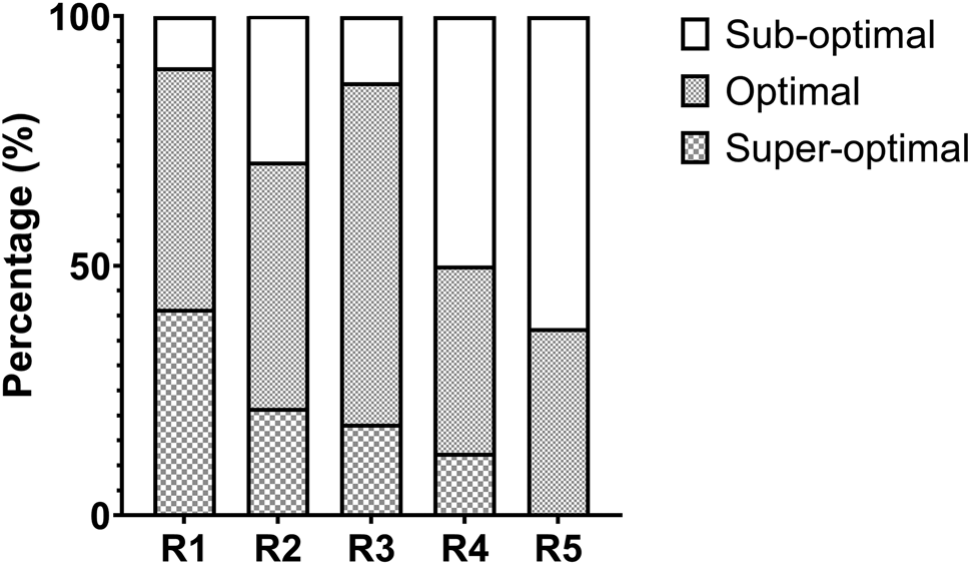
Distribution of PIFR groups among different DPI resistances. *PIFR* peak inspiratory flow rate, *DPI* dry powder inhaler.

The distribution of PIFR groups among the different DPI resistance groups is shown in Fig. 2. The percentage of superoptimal PIFR was highest in R1 (41.4%), followed by R2 (21.5%), R3 (18.4%), R4 (12.5%), and R5 (0.0%). On the contrary, the percentage of sub-optimal PIFR was lowest in R1 (10.2%), followed by R2 (29.2%), R3 (13.2%), R4 (50.0%), and R5 (62.5%).

In univariable multinomial logistic regression analysis, patients in the superoptimal PIFR group were more likely to be younger, male sex, higher BMI, and current smokers and to have less comorbidity, better lung function (FEV_1_%pred, FVC %pred, and diffusion capacity of carbon monoxide [DLCO] %pred), and fewer frequent exacerbations in the previous year compared to patients in the sub-optimal PIFR group (Table 2). The degree of association, presented as odds ratio (OR) with 95% confidence interval (CI), was more prominent in the superoptimal PIFR group than in the optimal PIFR group.

**Table 2:** Factors affecting PIFR using univariable multinomial logistic regression analysis.

	Sub-optimal	Optimal	*p*	Superoptimal	*p*	LR test
		OR (95% CI)		OR (95% CI)		
Age	Reference	0.96 (0.93–0.99)	0.008	0.92 (0.89–0.95)	< 0.001	< 0.001
Male	Reference	2.93 (1.30–6.59)	0.009	4.96 (1.58–15.59)	0.006	0.005
BMI	Reference	1.06 (0.98–1.14)	0.125	1.15 (1.06–1.25)	0.001	0.003
Smoking						0.036
Former	Reference	0.89 (0.45–1.73)	0.72	0.048 (0.24–0.96)	0.038	
Never	Reference	0.32 (0.09–1.13)	0.078	0.18 (0.04–0.81)	0.025	
CAT score	Reference	0.96 (0.93–0.99)	0.006	0.97 (0.93–1.00)	0.068	0.023
CCI score		0.87 (0.74–1.04)	0.119	0.59 (0.45–0.76)	< 0.001	< 0.001
FEV_1_%pred^a^	Reference	1.02 (1.01–1.04)	0.006	1.04 (1.02–1.06)	< 0.001	< 0.001
FVC %pred^a^	Reference	1.03 (1.01–1.04)	0.002	1.05 (1.03–1.07)	< 0.001	< 0.001
Post BD FEV_1_/FVC%	Reference	2.81 (0.39–20.14)	0.303	5.01 (0.53–47.02)	0.158	0.361
DLCO %pred	Reference	1.02 (1.00–1.03)	0.037	1.02 (1.01–1.04)	0.006	0.017
RV/TLC%	Reference	0.98 (0.95–1.01)	0.231	0.93 (0.89–0.97)	< 0.001	< 0.001
Frequent exacerbation^b^	Reference	0.55 (0.23–1.29)	0.167	0.22 (0.06–0.82)	0.024	0.048

*PIFR* peak inspiratory flow rate, *BMI* body mass index, *CAT* COPD assessment test, *mMRC* modified medical research council, *CCI* Charlson’s comorbidity index, *FEV*_*1*_ forced expiratory volume in 1 s, *FVC* forced vital capacity, *BD* bronchodilator, *DLCO* diffusion capacity, *RV* residual volume, *TLC* total lung capacity, *LR* likelihood ratio, *OR* odds ratio, *CI* confidence interval. *%pred* % of the predicted value.

^a^The value was obtained in post-bronchodilator spirometry.

^b^Frequent exacerbation was defined as ≥ 2 moderate or ≥ 1 severe exacerbation in the previous year.

The factors affecting the PIFR in the multivariate multinomial logistic regression model are presented in Table 3. Among the three PIFR groups, the superoptimal group had the youngest age, highest proportion of male sex, highest BMI, lowest CCI score, fewest frequent exacerbations in the previous year, and highest FVC %pred. However, no significant differences were observed between the optimal and sub-optimal PIFR groups, except for males.

**Table 3:** Factors affecting PIFR using multinomial multivariable logistic regression analyses.

	Sub-optimal	Optimal	*p*	Superoptimal	*p*	LR test
		OR (95% CI)		OR (95% CI)		
Age	Reference	0.97 (0.94–1.00)	0.067	0.94 (0.90–0.98)	0.003	0.012
Male	Reference	3.13 (1.22–8.01)	0.018	7.69 (1.91–31.06)	0.004	0.006
BMI	Reference	1.06 (0.97–1.16)	0.178	1.19 (1.08–1.33)	0.001	0.001
Smoking						0.325
Former	Reference	0.91 (0.44–1.89)	0.801	0.56 (0.25–1.26)	0.158	
Never	Reference	0.98 (0.23–4.22)	0.979	1.80 (0.28–11.71)	0.541	
CAT score	Reference	0.97 (0.94–1.00)	0.084	0.99 (0.95–1.04)	0.809	0.136
CCI score	Reference	0.88 (0.74–1.06)	0.19	0.59 (0.45–0.78)	< 0.001	< 0.001
Frequent exacerbation^b^	Reference	0.67 (0.26–1.74)	0.413	0.18 (0.03–0.99)	0.049	0.084
FEV_1_%pred^a^	Reference	1.01 (0.99–1.03)	0.48	1.01 (0.99–1.04)	0.458	0.728
FVC %pred^a^	Reference	1.02 (0.99–1.04)	0.112	1.05 (1.02–1.07)	< 0.001	0.002

*PIFR* peak inspiratory flow rate, *PFT* pulmonary function testing, *BMI* body mass index, *CAT* COPD assessment test, *CCI* Charlson comorbidity index, *FEV*_*1*_ forced expiratory volume in 1 s, *FVC* forced vital capacity, *LR* likelihood ratio, *OR* odds ratio, *CI* confidence interval. *%pred* % of the predicted value.

^a^The value was obtained in post-bronchodilator spirometry.

^b^Frequent exacerbation was defined as ≥ 2 moderate or ≥ 1 severe exacerbation in the previous year.

Subgroup analysis revealed prominent associations in terms of age, sex, BMI, frequent exacerbations in the previous year, and FVC %pred in COPD patients with more severe airflow limitation and FEV_1_ < 70%pred (Table 4). In addition, the superoptimal PIFR group was less likely to experience frequent exacerbations in the previous year than the sub-optimal PIFR group, but this relationship was only observed in patients with FEV_1_ < 70%pred (Fig. 1). Exacerbations in the previous year did not significantly differ between the sub-optimal and optimal PIFR groups.

**Table 4:** Subgroup analysis in by post-bronchodilator FEV_1_%pred of factors affecting PIFR using multinomial multivariable logistic regression analyses.

	Sub-optimal	Optimal	*p*	Superoptimal	*p*	LR test
FEV_1_%pred^a^ ≥ 70						
Age	Reference	0.99 (0.93–1.07)	0.87	1.00 (0.93–1.08)	0.964	0.979
Male	Reference	1.18 (0.10–13.67)	0.897	NA	NA	0.148
BMI	Reference	1.00 (0.84–1.19)	0.996	1.11 (0.92–1.33)	0.269	0.224
Smoking						0.078
Former	Reference	3.61 (1.10–11.80)	0.034	1.49 (0.45–4.98)	0.516	
Never		NA	NA	NA	NA	
CAT score	Reference	0.95 (0.87–1.03)	0.201	0.99 (0.91–1.08)	0.897	0.229
CCI score	Reference	0.75 (0.53–1.07)	0.11	0.42 (0.26–0.69)	0.001	< 0.001
Frequent exacerbation^b^	Reference	0.07 (0.00–1.94)	0.115	0.13 (0.00–3.90)	0.24	0.3
FVC %pred^a^	Reference	1.01 (0.97–1.06)	0.614	1.06 (1.01–1.11)	0.032	0.013
FEV_1_%pred^a^ < 70						
Age	Reference	0.96 (0.93–1.00)	0.067	0.91 (0.87–0.96)	0.001	0.002
Male	Reference	4.24 (1.35–13.29)	0.013	5.57 (1.16–26.79)	0.032	0.022
BMI	Reference	1.11 (1.01–1.22)	0.04	1.29 (1.14–1.47)	< 0.001	< 0.001
Smoking						0.346
Former	Reference	0.43 (0.16–1.14)	0.09	0.32 (0.10–1.02)	0.055	
Never		0.58 (0.11–3.18)	0.53	0.59 (0.06–5.81)	0.651	
CAT score	Reference	0.98 (0.94–1.02)	0.296	0.99 (0.95–1.04)	0.772	0.537
CCI score	Reference	0.95 (0.75–1.19)	0.646	0.75 (0.52–1.08)	0.117	0.175
Frequent exacerbation^b^	Reference	0.63 (0.22–1.79)	0.385	0.09 (0.01–0.83)	0.034	0.047
FVC %pred^a^	Reference	1.03 (1.00–1.05)	0.023	1.05 (1.02–1.08)	0.001	0.346

*PIFR* peak inspiratory flow rate, *PFT* pulmonary function testing, *BMI* body mass index, *CAT* COPD assessment test, *CCI* Charlson comorbidity index, *FEV*_*1*_ forced expiratory volume in 1 s, *FVC* forced vital capacity, *LR* likelihood ratio, *NA* not available. *%pred* % of the predicted value.

^a^The value was obtained in post-bronchodilator spirometry.

^b^Frequent exacerbation was defined as ≥ 2 moderate or ≥ 1 severe exacerbation in the previous year.

## Discussion

Using real-world clinical data of spirometry-confirmed COPD patients across seven tertiary hospitals in South Korea, we have demonstrated that COPD patients with superoptimal PIFR have different characteristics from those with optimal and sub-optimal PIFR. Among the three PIFR groups, COPD patients with superoptimal PIFR had the youngest age, highest proportion of male sex, highest BMI, lowest CCI score, least frequent exacerbations in the previous year, highest FVC %pred, and highest FEV_1_%pred. This association was more prominent in COPD patients with FEV_1_ < 70%pred than in those with ≥ 70%pred. In a real-world clinical setting, clinicians may gain additional insights into PIFR, considering various clinical characteristics, through the measurement of this value using devices such as the In-Check Dial.

Notably, frequent exacerbations in the previous year were fewest in the superoptimal PIFR group, whereas no differences were observed between the sub-optimal and optimal PIFR groups. This association was more prominent in COPD patients with FEV_1_ < 70%pred than it was in those with ≥ 70%pred. This result suggests that superoptimal PIFR is a distinguishing phenotype in COPD patients using DPI, with a lower probability of exacerbation. This is an extension of the observation that a superoptimal PIFR is closely associated with young age, male sex, higher BMI, lower CCI score, and higher FVC %pred. In line with our findings, previous studies have shown that frequent exacerbation in COPD is related to older age^11^, female sex^12^, lower BMI^13^, and higher comorbidities^14^, all of which imply a bundle of characteristics against the superoptimal PIFR.

Other factors associated with the superoptimal PIFR group were younger age, male sex, higher BMI, lower comorbidity burden, and higher FVC %pred. These factors were more closely related to superoptimal PIFR in COPD patients with FEV_1_ < 70%pred than in those with FEV_1_ ≥ 70%pred. Our result confirms prior findings that male sex and younger age were more likely to be associated with higher PIFR than female sex and older age were^1,3,15^, Given that inspiratory muscle strength depends on sex, age, and anthropometric indices, the observed finding in our study may be not surprising^16,17^, Respiratory muscle power, which was assessed using the maximum inspiratory pressure, was higher in obese individuals than in eutrophic individuals^16^. In terms of FVC %pred, a weak but significant correlation (r = 0.37, *p* < 0.001) with PIFR has been reported^3^, which is similar to our results (r = 0.316, *p* < 0.001) obtained from a Pearson’s correlation analysis. In COPD patients with FEV_1_ ≥ 70%pred, the airway obstruction may not be sufficiently severe to create a significant effect on PIFR or cause notable differences related to clinical factors, such as BMI or sex. Consequently, the observed difference according to the FEV_1_%pred suggests that superoptimal PIFR in COPD patients with severe airflow limitation can be a favorable trait. In contrast, a sub-optimal PIFR may represent a treatable trait. Notably, inspiratory muscle training increases PIFR in patients with severe COPD^18^. This finding may have clinical implications, suggesting that patients with severe COPD who are unable to achieve an optimal PIFR against DPI may significantly benefit from inspiratory muscle training and that this may represent a treatable trait^19^.

Our study suggests that superoptimal PIFR may be considered as another phenotype of COPD patients who are using DPI, although further longitudinal studies are necessary. Superoptimal, excessive PIFR, is often regarded as inappropriate for optimal drug delivery to the lung^7–9^. A previous study showed that a faster inspiratory flow (> 60 L/min) decreased particle deposition in the lungs and increased oropharyngeal deposition^9^. Another study in children with asthma suggested an optimal PIFR range, showing similar clinical outcomes within a range between 30 L/min and 60 L/min or 90 L/min of PIFR for Turbohaler and Diskus, respectively^20^. The concept that there is a maximal value of proper PIFR is based on the observation that more oropharyngeal deposition is related to faster PIFR^21^. However, the actual mean values of the PIFRs for Turbohaler and Diskus were 82.8 L/min and 105.6 L/min, respectively^21^. Similarly, another study showed that the mean PIFR against the R1 device was approximately 80 L/min^4^, and a high proportion of PIFR > 90 L/min was observed in the low-resistance device, which is consistent with our findings. Therefore, considering the heterogeneity within the PIFR group, formerly uniformly categorized as the optimal PIFR group^2–5,21^, and the difference in clinical characteristics among the sub-optimal, optimal, and superoptimal PIFR groups, further studies are warranted to elucidate the longitudinal effects of superoptimal PIFR in COPD patients. In the additional subgroup analysis performed for patients with a superoptimal PIFR according to the FEV_1_% pred and PIFR, the residual volume/total lung capacity (RV/TLC%) was lower in those with a PIFR ≥ 100 L/min than in those with a PIFR < 100 L/min, irrespective of the FEV_1_%pred (Table S1). Additional studies may provide insights into the physiological factors underlying the negative correlation between the PIFR and RV/TLC%. For instance, it would be helpful to measure the total lung capacity across more patients, analyse small and large airway abnormalities using other techniques such as computed tomography or oscillometry, and verify conditions linked to the inspiratory strength (such as muscle strength)^22,23^. It may also be the case that superoptimal PIFR reflects the individuals underlying fitness and thus the effects of delivery of inhaled medication to the lungs becomes less relevant. However, we feel that this would lead to the potential for both effects to cancel each other out: less efficient delivery of drug and better underlying health status.

Our study had some limitations. First, as this was a cross-sectional study, the results should be interpreted with caution. There was a lack of temporality, and causal relationship was not explained. For example, it is inappropriate to conclude that a superoptimal PIFR is beneficial for the future risk of exacerbation. Further longitudinal studies are required to differentiate the clinical course of COPD patients with superoptimal PIFR. Second, there were no data on eosinophil counts or use of inhaled corticosteroids. Given the close relationship among blood eosinophil count, maintenance device therapy, and exacerbation^24^, the application of these factors could alter the observed findings. Third, only the In-Check Dial was used to assess PIFR and categorize the patients into PIFR groups. Although using this device is a popular way to evaluate patients’ ability to generalize inspiratory flows, considering other parameters, such as pressure drop, would provide a more relevant way to optimize the DPI device^25^. Also, the assessment of PIFR does not consider inspiratory duration which also has to be adequate to enable effective deposition of treatment into the lungs from a DPI. Finally, although the In-Check Dial has a red-colorized boundary indicating the upper optimal value as 90 L/min, and we utilized the cut-off value of 90 L/min in accordance with previous reports^7,8^, it is important to acknowledge that this might is arbitrary and will be affected by the intrinsic resistance of the inhaler device and should be validated in future studies.

In conclusion, superoptimal PIFR can be another phenotype with characteristics different from those of the optimal and sub-optimal PIFR groups. In particular, patients in the superoptimal PIFR group are more likely to be younger and men and have higher BMI, lower comorbidities, fewer frequent exacerbations in the previous year, and higher FVC %pred. This is more pronounced in COPD patients with FEV_1_ < 70%pred than in those with predicted FEV_1_ ≥ 70%pred, suggesting that superoptimal PIFR may be a favorable trait in severe COPD and encouraging patients in the sub-optimal PIFR group to receive inspiratory muscle training to improve their PIFR. In a real clinical practice, by measuring the PIFR using devices such as the In-Check Dial, clinicians may gain additional insights into PIFR, considering various clinical characteristics. Further longitudinal studies are necessary to identify the clinical course of COPD patients with superoptimal PIFR.

## Methods

### Study design and patients

This multi-center cross-sectional study was conducted in seven tertiary hospitals in South Korea. COPD patients were recruited between June 2021 and November 2021 to evaluate their PIFR who met the following inclusion criteria: (1) aged ≥ 40 years, (2) diagnosis of COPD by post-bronchodilator ratio of FEV_1_/FVC < 0.7^26^, (3) treatment with DPI > 3 months before the recruitment, and (4) regular outpatient visit. During the recruitment process, COPD patients with the following conditions were excluded: (1) patients with a history of asthma or asthma–COPD overlap, (2) patients receiving home oxygen therapy, (3) patients with significant morphological underlying lung diseases such as tuberculosis-destroyed lung or bronchiectasis, and (4) patients with a recent history of severe cardiovascular disease or end-stage cancer. Ultimately, 444 COPD patients were identified.

The study protocol was approved by the Institutional Review Board of Ulsan University Hospital (no. 2019-07-038). This study was conducted following the Declaration of Helsinki. All procedures were performed in accordance with relevant guidelines and regulations.

### Groups according to PIFR

The PIFR (L/min) generated in the presence of different inhalational resistances was measured using an In-Check Dial G16 (Clement Clarke, UK). The In-Check Dial G16 can be set to the intrinsic resistance of the inhaler that the patient uses. The patients were instructed to fully exhale and then inhale as hard and as fast as possible. The maximum PIFR was obtained during three attempts. The maximum PIFR for each device was recorded separately.

The resistance values evaluated were categorized as low (R1, representing Breezhaler), low-medium (R2, representing Ellipta and Diskus), medium (R3, representing Turbohaler Symbicort and Genuair), medium–high (R4, representing Nexthaler), and high (R5, representing Handihaler)^4,27^.

Sub-optimal PIFR was defined if In-Check Dial measurements were with any resistance range (< 50, < 60, < 60 [or < 45 for Genuair], < 35, and < 30 with R1, R2, R3, R4, and R5, respectively)^4,27^. Superoptimal PIFR was defined as having a maximum PIFR of ≥ 90 L/min from any utilized device^7,8^. The optimal PIFR was neither sub-optimal nor superoptimal.

### Variables

The most recent values of pulmonary function test measured within 3 months of recruitment were collected. Both pre- and post-bronchodilator results were collected. Data on FEV_1_ (L, % pred), FVC (L, % pred), and FEV_1_/FVC (%) were collected. Data on DLCO and residual volume and total lung capacity were available for 301 and 172 patients, respectively.

Exacerbation history in the year prior to recruitment was also collected. A moderate exacerbation was defined as an outpatient visit with a prescription of antibiotics or systemic glucocorticoids. Severe exacerbations were defined as patient visits to the emergency room or requirement of hospitalization because of exacerbation. We classified the presence of frequent exacerbation event as having ≥ 2 moderate or ≥ 1 severe history in the previous year^26^.

Electronic medical records were reviewed to collect the following variables: age, sex, height (cm), weight (kg), BMI (kg/m^2^), smoking status (never, former, and current), CAT score, and comorbidities to calculate the CCI score^28^.

### Statistical analysis

Comparisons of clinical variables among the PIFR groups were performed using one-way analysis of variance for continuous variables and the chi-squared or Fisher’s exact test for categorical variables. Bonferroni correction was used for post-hoc analysis, and the threshold for significance was determined at *p* = 0.017 (0.05/3). A multinomial logistic regression analysis was performed to determine the clinical factors related to PIFR. ORs and 95% CIs were calculated. Factors with *p* < 0.1 in univariable analysis were considered for the multivariable model. A likelihood ratio test was used to estimate the model’s goodness of fit. Subgroup analysis was performed stratified by post-bronchodilator FEV_1_% pred (≥ 70 and < 70). An additional subgroup analysis was performed among patients with a superoptimal PIFR according to the median FEV_1_%pred and PIFR to identify any differences within this group. We determined the 70% for cut-off as the median value. All statistical analyses were performed using the SPSS (version 25 for Windows, Chicago, IL, USA) and R software version 4.3.1 for Windows (R Development Core Team). Statistical significance was set at *p* < 0.05.

### Ethical approval

Institutional Review Board of Ulsan University Hospital (no. 2019-07-038) approved the study protocol and waived the informed consent from the participants since the nature of this study was retrospective and patient data were anonymized. This study was conducted in accordance with the Declaration of Helsinki. All procedures were performed in accordance with the relevant guidelines and regulations.

### Supplementary Information

Supplementary Table S1.

## Data Availability

The data that support the findings of this study are available from the corresponding author upon reasonable request.
