# Supplementary material for: Clinical characteristics of chronic obstructive pulmonary disease patients with superoptimal peak inspiratory flow rate
Source: Sci Rep. 2024 Jul 3;14:15337. doi: 10.1038/s41598-024-65085-2 (PMC11222384; doi:10.1038/s41598-024-65085-2)
Supplement: Supplementary file 1 — Supplementary Table S1. [file 41598_2024_65085_MOESM1_ESM.docx]

**Table S1.** Characteristics of COPD patients with super-optimal PIFR according to their PIFR and FEV_1_% pred.

|  | **PIFR <100 &**  **FEV_1_% pred <70**  **(G1, n=24)** | **PIFR** **≥100 &**  **FEV_1_% pred <70**  **(G2, n=37)** | **PIFR <100 &**  **FEV_1_% pred ≥70**  **(G3, n=18)** | **PIFR ≥100 &**  **FEV_1_% pred ≥70**  **(G4, n=44)** | ***p*** |
| --- | --- | --- | --- | --- | --- |
| **Age** (years) | 66.7 (6.1) | 69.3 (8.4) | 71.6 (8.1) | 69.4 (7.0) | 0.248 |
| **Male**, n (%) | 22 (91.7) | 35 (94.6) | 18 (100.0) | 44 (100.0) | 0.21 |
| **BMI** (kg/m^2^) | 24.2 (3.7) | 24.5 (2.7) | 24.1 (2.3) | 24.5 (2.6) | 0.946 |
| **Smoking**, n (%) |  |  |  |  | 0.176 |
| Never | 2 (8.3) | 0 (0.0) | 2 (5.9) | 0 (0.0) |  |
| Former | 17 (70.8) | 28 (75.7) | 9 (52.9) | 33 (75.0) |  |
| Current | 5 (20.8) | 9 (24.3) | 7 (41.2) | 11 (25.0) |  |
| **CAT score** | 9.1 (7.9) | 11.6 (7.8) | 6.9 (5.3) | 10.0 (6.5) | 0.156 |
| **CCI score** | 1.1 (0.9) | 1.0 (1.1) | 1.2 (0.7) | 0.8 (0.9) | 0.432 |
| **FEV_1_ %pred**^a)^ | 53.9 (12.1) | 56.7 (11.5) | 79.6 (8.3)†¶ | 83.6 (10.3)‡§ | <0.001 |
| **FVC %pred**^a)^ | 76.8 (16.5) | 80.0 (13.7) | 94.1 (15.2)†¶ | 95.1 (11.3)‡§ | <0.001 |
| **Post BD FEV_1_/FVC%** | 51.3 (9.2) | 50.2 (11.1) | 59.1 (6.5)†¶ | 61.6 (6.2)‡§ | <0.001 |
| **DLCO %pred** | 68.5 (22.8) | 65.5 (19.4) | 79.6 (20.1) | 70.4 (19.2) | 0.171 |
| **RV/TLC%** | 48.1 (14.2) | 41.0 (10.5)* | 40.0 (4.6)† | 31.6 (6.2) ‡§# | <0.001 |
| **Highest PIFR** (L/min) | 90.9 (1.9) | 111.8 (9.0)* | 91.5 (2.3)¶ | 112.7 (8.2)‡# | <0.001 |
| **Frequent exacerbation^b)^**, n (%) | 0 (0.0) | 1 (2.7) | 0 (0.0) | 2 (4.5) | 0.606 |

Continuous and categorical variables are presented as means with standard deviations and numbers with percentages, respectively.

a) The value was obtained in post-bronchodilator spirometry

b) Frequent exacerbation was defined as ≥2 moderate or ≥1 severe exacerbation in the previous year.

* *p*<0.05 G1 versus G2 in post-hoc analysis with Bonferroni correction.

† *p*<0. 05 G1 versus G3 in post-hoc analysis with Bonferroni correction.

‡ *p*<0.05 G1 versus G4 in post-hoc analysis with Bonferroni correction.

¶ *p*<0.05 G2 versus G3 in post-hoc analysis with Bonferroni correction.

§ *p*<0.05 G2 versus G4 in post-hoc analysis with Bonferroni correction.

# *p*<0.05 G3 versus G4 in post-hoc analysis with Bonferroni correction.

COPD; chronic obstructive pulmonary disease, PIFR; peak inspiratory flow rate, BMI; body mass index, CAT; COPD assessment test, mMRC; modified medical research council, CCI; Charlson comorbidity index, FEV_1_; forced expiratory volume in 1 s, FVC; forced vital capacity, BD; bronchodilator, DLCO; diffusion capacity, RV; residual volume, TLC; total lung capacity. % pred; % of the predicted value.
